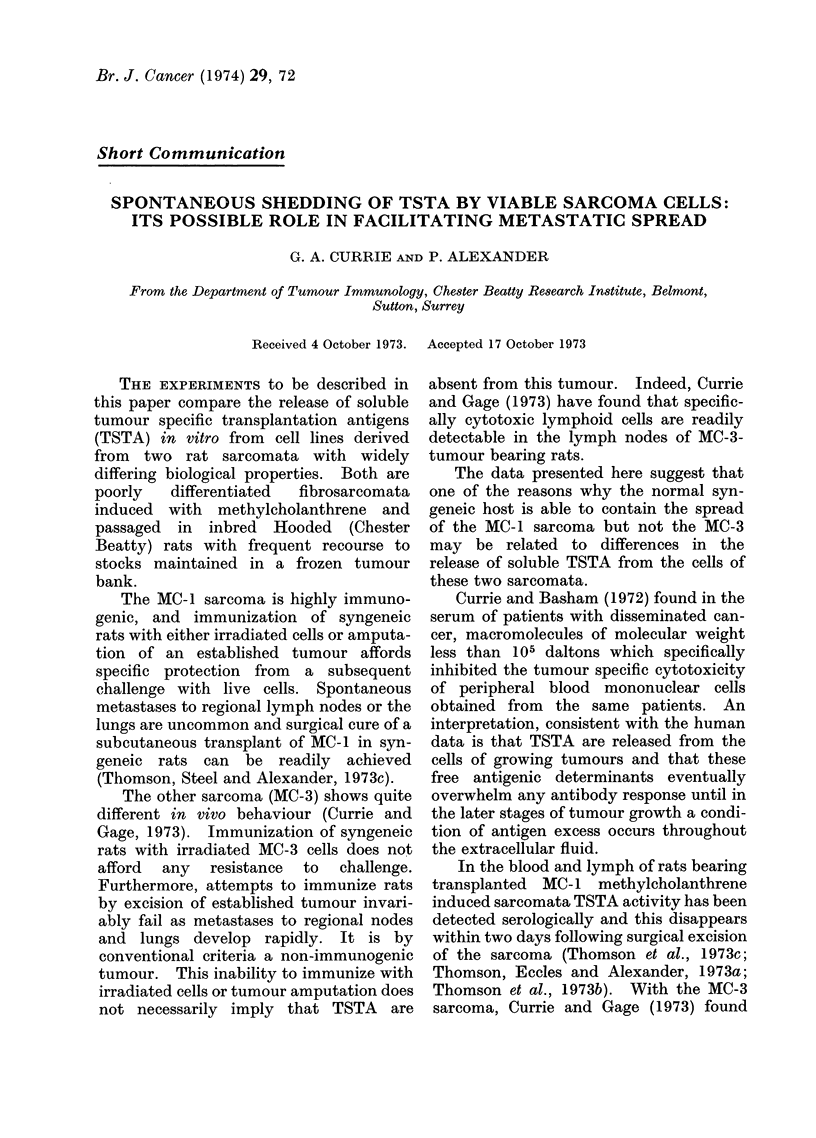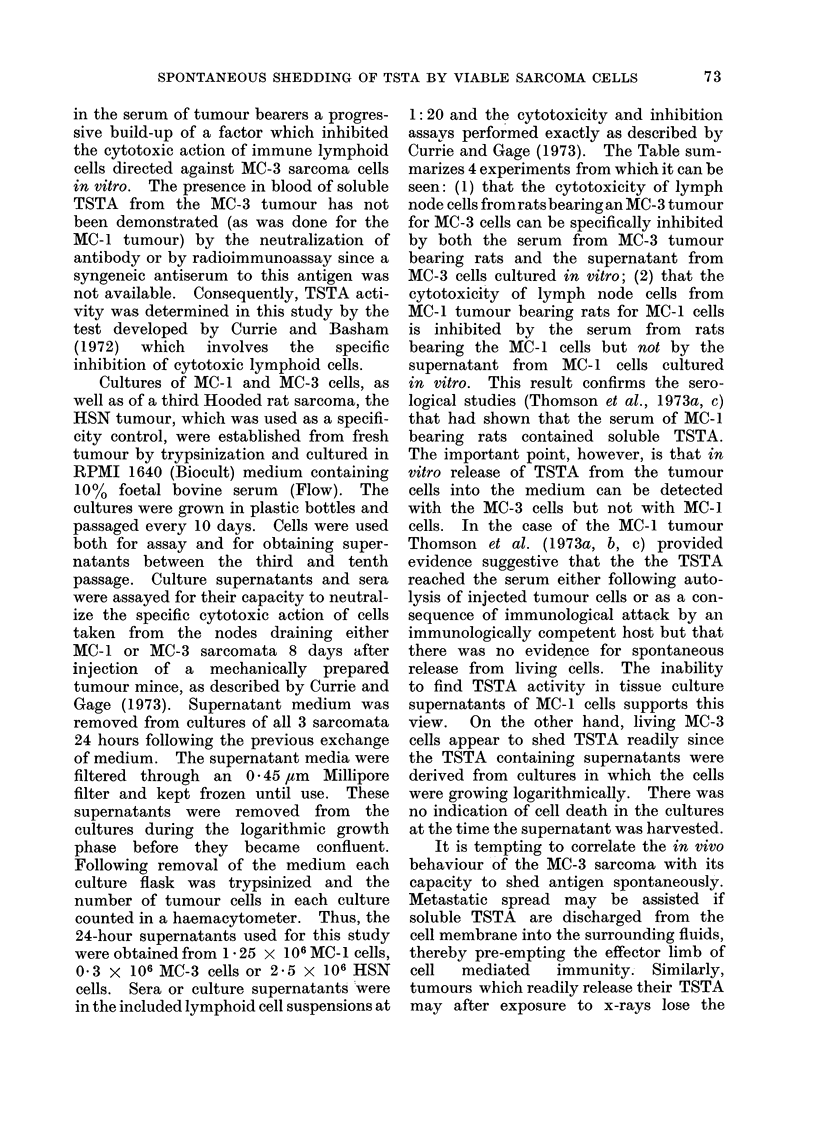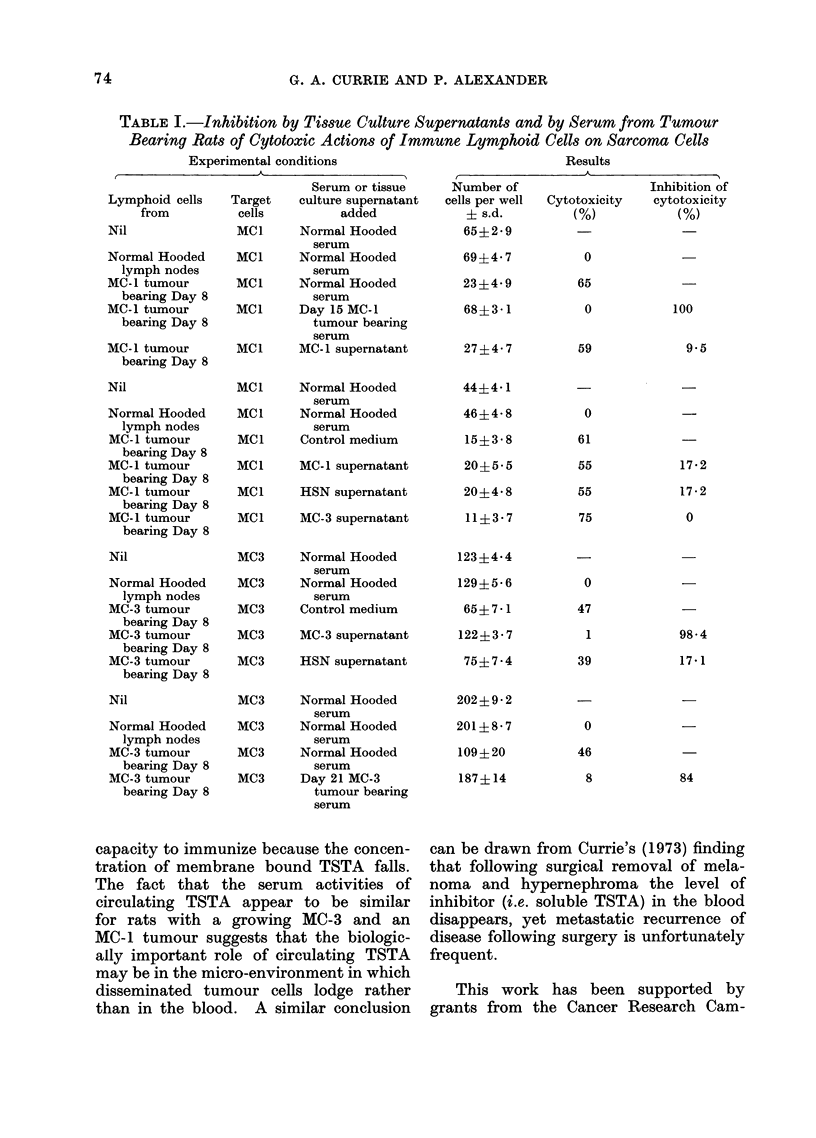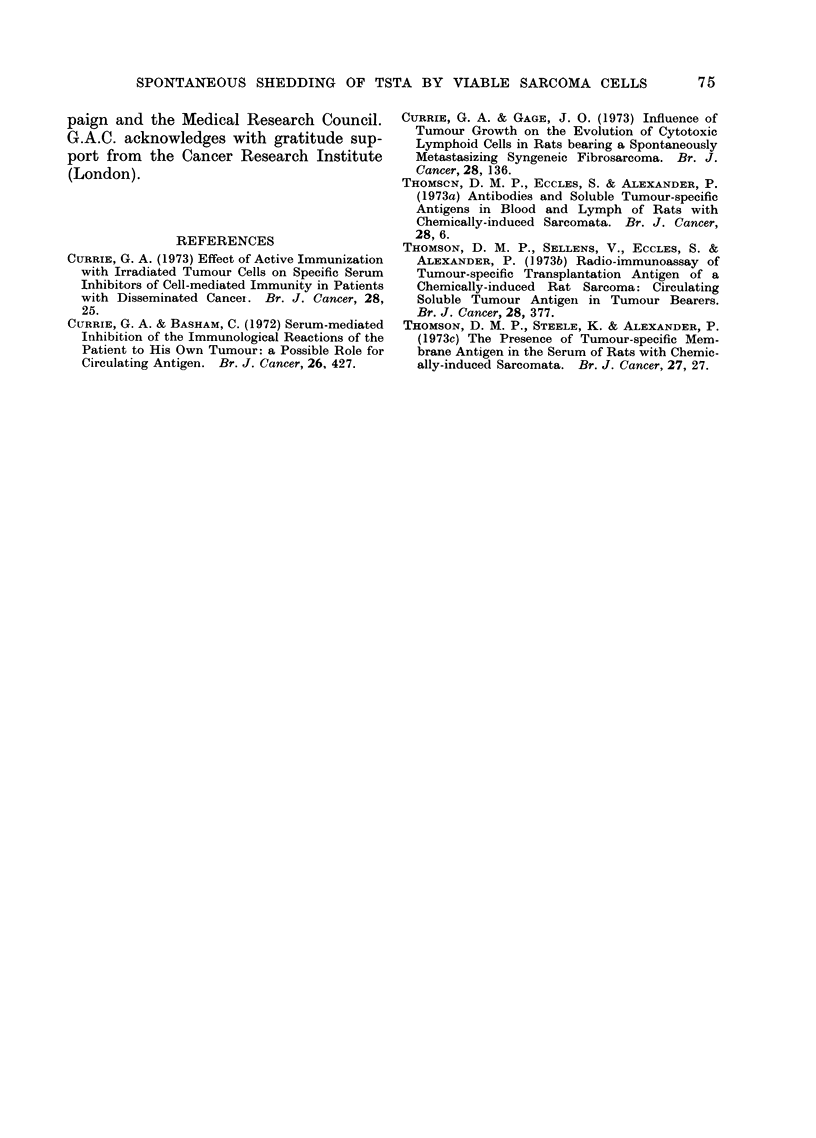# Spontaneous shedding of TSTA by viable sarcoma cells: its possible role in facilitating metastatic spread.

**DOI:** 10.1038/bjc.1974.9

**Published:** 1974-01

**Authors:** G. A. Currie, P. Alexander


Br. J. Cancer (1974) 29, 72

### Short Communication

### SPONTANEOUS SHEDDING OF TSTA BY VIABLE SARCOMA CELLS: ITS POSSIBLE ROLE IN FACILITATING METASTATIC SPREAD

G. A. CURRIE AND P. ALEXANDER

*From the Department of Tumour Immunology, Chester Beatty Research Institute, Belmont, Sutton, Surrey*

Received 4 October 1973. Accepted 17 October 1973

THE EXPERIMENTS to be described in this paper compare the release of soluble tumour specific transplantation antigens (TSTA) *in vitro* from cell lines derived from two rat sarcomata with widely differing biological properties. Both are poorly differentiated fibrosarcomata induced with methylcholanthrene and passaged in inbred Hooded (Chester Beatty) rats with frequent recourse to stocks maintained in a frozen tumour bank.

The MC-1 sarcoma is highly immunogenic, and immunization of syngeneic rats with either irradiated cells or amputation of an established tumour affords specific protection from a subsequent challenge with live cells. Spontaneous metastases to regional lymph nodes or the lungs are uncommon and surgical cure of a subcutaneous transplant of MC-1 in syngeneic rats can be readily achieved (Thomson, Steel and Alexander, 1973c).

The other sarcoma (MC-3) shows quite different *in vivo* behaviour (Currie and Gage, 1973). Immunization of syngeneic rats with irradiated MC-3 cells does not afford any resistance to challenge. Furthermore, attempts to immunize rats by excision of established tumour invariably fail as metastases to regional nodes and lungs develop rapidly. It is by conventional criteria a non-immunogenic tumour. This inability to immunize with irradiated cells or tumour amputation does not necessarily imply that TSTA are absent from this tumour. Indeed, Currie and Gage (1973) have found that specifically cytotoxic lymphoid cells are readily detectable in the lymph nodes of MC-3-tumour bearing rats.

The data presented here suggest that one of the reasons why the normal syngeneic host is able to contain the spread of the MC-1 sarcoma but not the MC-3 may be related to differences in the release of soluble TSTA from the cells of these two sarcomata.

Currie and Basham (1972) found in the serum of patients with disseminated cancer, macromolecules of molecular weight less than $10^5$ daltons which specifically inhibited the tumour specific cytotoxicity of peripheral blood mononuclear cells obtained from the same patients. An interpretation, consistent with the human data is that TSTA are released from the cells of growing tumours and that these free antigenic determinants eventually overwhelm any antibody response until in the later stages of tumour growth a condition of antigen excess occurs throughout the extracellular fluid.

In the blood and lymph of rats bearing transplanted MC-1 methylcholanthrene induced sarcomata TSTA activity has been detected serologically and this disappears within two days following surgical excision of the sarcoma (Thomson *et al.*, 1973*c*; Thomson, Eccles and Alexander, 1973*a*; Thomson *et al.*, 1973*b*). With the MC-3 sarcoma, Currie and Gage (1973) found in the serum of tumour bearers a progressive build-up of a factor which inhibited the cytotoxic action of immune lymphoid cells directed against MC-3 sarcoma cells *in vitro*. The presence in blood of soluble TSTA from the MC-3 tumour has not been demonstrated (as was done for the MC-1 tumour) by the neutralization of antibody or by radioimmunoassay since a syngeneic antiserum to this antigen was not available. Consequently, TSTA activity was determined in this study by the test developed by Currie and Basham (1972) which involves the specific inhibition of cytotoxic lymphoid cells.

Cultures of MC-1 and MC-3 cells, as well as of a third Hooded rat sarcoma, the HSN tumour, which was used as a specificity control, were established from fresh tumour by trypsinization and cultured in RPMI 1640 (Biocult) medium containing 10% foetal bovine serum (Flow). The cultures were grown in plastic bottles and passaged every 10 days. Cells were used both for assay and for obtaining supernatants between the third and tenth passage. Culture supernatants and sera were assayed for their capacity to neutralize the specific cytotoxic action of cells taken from the nodes draining either MC-1 or MC-3 sarcomata 8 days after injection of a mechanically prepared tumour mince, as described by Currie and Gage (1973). Supernatant medium was removed from cultures of all 3 sarcomata 24 hours following the previous exchange of medium. The supernatant media were filtered through an $0 \cdot 45\ \mu m$ Millipore filter and kept frozen until use. These supernatants were removed from the cultures during the logarithmic growth phase before they became confluent. Following removal of the medium each culture flask was trypsinized and the number of tumour cells in each culture counted in a haemacytometer. Thus, the 24-hour supernatants used for this study were obtained from $1 \cdot 25 \times 10^6$ MC-1 cells, $0 \cdot 3 \times 10^6$ MC-3 cells or $2 \cdot 5 \times 10^6$ HSN cells. Sera or culture supernatants were in the included lymphoid cell suspensions at 1:20 and the cytotoxicity and inhibition assays performed exactly as described by Currie and Gage (1973). The Table summarizes 4 experiments from which it can be seen: (1) that the cytotoxicity of lymph node cells from rats bearing an MC-3 tumour for MC-3 cells can be specifically inhibited by both the serum from MC-3 tumour bearing rats and the supernatant from MC-3 cells cultured *in vitro*; (2) that the cytotoxicity of lymph node cells from MC-1 tumour bearing rats for MC-1 cells is inhibited by the serum from rats bearing the MC-1 cells but *not* by the supernatant from MC-1 cells cultured *in vitro*. This result confirms the serological studies (Thomson *et al.*, 1973*a*, *c*) that had shown that the serum of MC-1 bearing rats contained soluble TSTA. The important point, however, is that *in vitro* release of TSTA from the tumour cells into the medium can be detected with the MC-3 cells but not with MC-1 cells. In the case of the MC-1 tumour Thomson *et al.* (1973*a*, *b*, c) provided evidence suggestive that the the TSTA reached the serum either following autolysis of injected tumour cells or as a consequence of immunological attack by an immunologically competent host but that there was no evidence for spontaneous release from living cells. The inability to find TSTA activity in tissue culture supernatants of MC-1 cells supports this view. On the other hand, living MC-3 cells appear to shed TSTA readily since the TSTA containing supernatants were derived from cultures in which the cells were growing logarithmically. There was no indication of cell death in the cultures at the time the supernatant was harvested.

It is tempting to correlate the *in vivo* behaviour of the MC-3 sarcoma with its capacity to shed antigen spontaneously. Metastatic spread may be assisted if soluble TSTA are discharged from the cell membrane into the surrounding fluids, thereby pre-empting the effector limb of cell mediated immunity. Similarly, tumours which readily release their TSTA may after exposure to x-rays lose the

TABLE I.—*Inhibition by Tissue Culture Supernatants and by Serum from Tumour Bearing Rats of Cytotoxic Actions of Immune Lymphoid Cells on Sarcoma Cells*

| Experimental conditions | | | Results | | |
|---|---|---|---|---|---|
| Lymphoid cells from | Target cells | Serum or tissue culture supernatant added | Number of cells per well ± s.d. | Cytotoxicity (%) | Inhibition of cytotoxicity (%) |
| Nil | MC1 | Normal Hooded serum | 65±2·9 | — | — |
| Normal Hooded lymph nodes | MC1 | Normal Hooded serum | 69±4·7 | 0 | — |
| MC-1 tumour bearing Day 8 | MC1 | Normal Hooded serum | 23±4·9 | 65 | — |
| MC-1 tumour bearing Day 8 | MC1 | Day 15 MC-1 tumour bearing serum | 68±3·1 | 0 | 100 |
| MC-1 tumour bearing Day 8 | MC1 | MC-1 supernatant | 27±4·7 | 59 | 9·5 |
| Nil | MC1 | Normal Hooded serum | 44±4·1 | — | — |
| Normal Hooded lymph nodes | MC1 | Normal Hooded serum | 46±4·8 | 0 | — |
| MC-1 tumour bearing Day 8 | MC1 | Control medium | 15±3·8 | 61 | — |
| MC-1 tumour bearing Day 8 | MC1 | MC-1 supernatant | 20±5·5 | 55 | 17·2 |
| MC-1 tumour bearing Day 8 | MC1 | HSN supernatant | 20±4·8 | 55 | 17·2 |
| MC-1 tumour bearing Day 8 | MC1 | MC-3 supernatant | 11±3·7 | 75 | 0 |
| Nil | MC3 | Normal Hooded serum | 123±4·4 | — | — |
| Normal Hooded lymph nodes | MC3 | Normal Hooded serum | 129±5·6 | 0 | — |
| MC-3 tumour bearing Day 8 | MC3 | Control medium | 65±7·1 | 47 | — |
| MC-3 tumour bearing Day 8 | MC3 | MC-3 supernatant | 122±3·7 | 1 | 98·4 |
| MC-3 tumour bearing Day 8 | MC3 | HSN supernatant | 75±7·4 | 39 | 17·1 |
| Nil | MC3 | Normal Hooded serum | 202±9·2 | — | — |
| Normal Hooded lymph nodes | MC3 | Normal Hooded serum | 201±8·7 | 0 | — |
| MC-3 tumour bearing Day 8 | MC3 | Normal Hooded serum | 109±20 | 46 | — |
| MC-3 tumour bearing Day 8 | MC3 | Day 21 MC-3 tumour bearing serum | 187±14 | 8 | 84 |

capacity to immunize because the concentration of membrane bound TSTA falls. The fact that the serum activities of circulating TSTA appear to be similar for rats with a growing MC-3 and an MC-1 tumour suggests that the biologically important role of circulating TSTA may be in the micro-environment in which disseminated tumour cells lodge rather than in the blood. A similar conclusion can be drawn from Currie's (1973) finding that following surgical removal of melanoma and hypernephroma the level of inhibitor (*i.e.* soluble TSTA) in the blood disappears, yet metastatic recurrence of disease following surgery is unfortunately frequent.

This work has been supported by grants from the Cancer Research Campaign and the Medical Research Council. G.A.C. acknowledges with gratitude support from the Cancer Research Institute (London).

### REFERENCES

CURRIE, G. A. (1973) Effect of Active Immunization with Irradiated Tumour Cells on Specific Serum Inhibitors of Cell-mediated Immunity in Patients with Disseminated Cancer. *Br. J. Cancer*, **28**, 25.

CURRIE, G. A. & BASHAM, C. (1972) Serum-mediated Inhibition of the Immunological Reactions of the Patient to His Own Tumour: a Possible Role for Circulating Antigen. *Br. J. Cancer*, **26**, 427.

CURRIE, G. A. & GAGE, J. O. (1973) Influence of Tumour Growth on the Evolution of Cytotoxic Lymphoid Cells in Rats bearing a Spontaneously Metastasizing Syngeneic Fibrosarcoma. *Br. J. Cancer*, **28**, 136.

THOMSCN, D. M. P., ECCLES, S. & ALEXANDER, P. (1973*a*) Antibodies and Soluble Tumour-specific Antigens in Blood and Lymph of Rats with Chemically-induced Sarcomata. *Br. J. Cancer*, **28**, 6.

THOMSON, D. M. P., SELLENS, V., ECCLES, S. & ALEXANDER, P. (1973*b*) Radio-immunoassay of Tumour-specific Transplantation Antigen of a Chemically-induced Rat Sarcoma: Circulating Soluble Tumour Antigen in Tumour Bearers. *Br. J. Cancer*, **28**, 377.

THOMSON, D. M. P., STEELE, K. & ALEXANDER, P. (1973*c*) The Presence of Tumour-specific Membrane Antigen in the Serum of Rats with Chemically-induced Sarcomata. *Br. J. Cancer*, **27**, 27.